# *G6PD* genetic variations in neonatal Hyperbilirubinemia in Indonesian Deutromalay population

**DOI:** 10.1186/s12887-019-1882-z

**Published:** 2019-12-20

**Authors:** Dewi A. Wisnumurti, Yunia Sribudiani, Robert M. Porsch, Ani M. Maskoen, Sri E. Rahayuningsih, Eni K. Asni, Frank Sleutels, Wilfred F. J. van Ijcken, Abdurachman Sukadi, Tri H. Achmad

**Affiliations:** 1 Departement of Pediatric, Neonatology Subdivision, Arifin Achmad General Hospital, Universitas Riau, Pekanbaru, Indonesia; 2 Research Center of Medical Genetics, Faculty of Medicine, Universitas Padjadjaran, Bandung, Indonesia; 3 Department of Biomedical Sciences, Division of Biochemistry and Molecular Biology, Faculty of Medicine, Universitas Padjadjaran, Bandung, Indonesia; 4 Department of Psychiatry, Li Ka Shing Faculty of Medicine, The University of Hong Kong, Hong kong SAR, China; 5 Departement of Pediatric, Cardiology Subdivision, Dr. Hasan Sadikin Hospital, Faculty of Medicine, Universitas Padjadjaran, Bandung, Indonesia; 6 Department of Biochemistry, Faculty of Medicine, Universitas Riau, Pekanbaru, Indonesia; 7 Erasmus Center for Biomics, Erasmus MC, Rotterdam, The Netherlands; 8 Departement of Pediatric, Neonatology Subdivision, Dr. Hasan Sadikin Hospital, Faculty of Medicine, Universitas Padjadjaran, Bandung, Indonesia

**Keywords:** Deutromalay, G6PD deficiency, Genetics variation, Neonatal Hyperbilirubinemia

## Abstract

**Background:**

Neonatal jaundice is a common finding in newborns in Asia, including Indonesia. In some cases, the serum total bilirubin levels exceeds the 95th percentile for hours of life (neonatal hyperbilirubinemia). Severe neonatal hyperbilirubinemia (NH) could lead to kernicterus and neonatal death. *Glucose-6-Phosphage Dehydrogenase* (*G6PD*) *g*enetic variations and deficiency have been reported in several studies to be associated with NH. This study aimed to analyze the *G6PD* genetic variations and its activity in neonates with and without hyperbilirubinemia in the Deutromalay Indonesian population.

**Methods:**

Deoxyribose Nucleic Acid (DNA) was isolated from peripheral blood of 116 and 115 healthy term neonates with and without hyperbilirubinemia. All infants underwent the following laboratory examinations: routine hematologic evaluation, Coombs test, G6PD activity measurement using the Randox kit method, and serum total bilirubin level. All exons of the *G6PD* gene were targeted for deep sequencing using MiSeq (Illumina). An association study of *G6PD* polymorphisms with NH was performed using PLINK.

**Results:**

The prevalence of G6PD deficiency in neonates with and without hyperbilirubinemia in Indonesian Deutromalay population were 1.72% (95% Confidence Interval (CI): 0.6–4.1%) and 1.74% (95% CI: 0.7–4.1%), respectively. The most common *G6PD* polymorphisms, i.e. rs1050757/c.* + 357A > G, rs2230037/c.1311C > T, and rs2071429/c.1365-13 T/IVS11, were identified. However, none of those polymorphisms and their haplotype were associated with NH (*p* > 0.05, Odds Ratio (OR) ~1.00). The prevalence of *G6PD* mutations in neonates with and without hyperbilirubinemia were 6.8% (95% CI: 2.3–11.5%) and 6.9% (95% CI: 2.3–11.6%), respectively. The most frequently identified *G6PD* mutation was the Viangchan variant (p.V291 M), which was followed by the Canton (p.R459L) and Vanua Lava (p.L128P) variants. Two novel mutations were identified both in case (p.V369A, p.I167F) and control (p.L474=, p.I36T) groups.

**Conclusion:**

The prevalence of G6PD deficiency is low in neonates with or without hyperbilirubinemia in Deutromalay Indonesian population. The majority of *G6PD* mutations identified among Indonesian Deutromalay population in this study are Viangchan, Canton and Vanua Lava variants.

## Background

*Glucose-6-phosphate-dehydrogenase* (*G6PD*) is a “housekeeping” gene encoding the G6PD enzyme which catalyzes glucose-6-phoshpate conversion to 6-phosphogluconolactone in the pentose monophosphate pathways in all cells [1, 2]. This enzyme is also important for maintaining red blood cells (RBCs) and protecting them from damages or premature destruction caused by oxidative stress through the maintenance of Nicotinamide Adenine Dinucleotide (NADP) and Nicotinamide Adenine Dinucleotide Phosphate Hydrogen (NADPH) levels [1, 2]. Although G6PD deficiency affects all cells in the body, the most affected cells are RBCs because these cells have no alternative pathways to produce NADPH [1]. The G6PD gene, 18 k base (kb) long and is located on chromosome Xq28, consists of 13 exons and 12 introns. The complete coding sequence is 1548 base pair (bp) long and encodes 514 amino acids [1, 3, 4]. Mutations throughout the *G6PD* gene lead to a deficiency in protein functions. Based on their biochemical and physicochemical characteristics, over 400 variants of G6PD have been reported. However, based on the type of mutations, those protein variants resulted from only ~140 different mutations [3–5]. Glucose-6-phosphate-dehydrogenase deficiency is the most common pentose monophosphate pathway enzyme deficiency that has been reported to affect 400 million people globally with the highest incidence in African, Mediterranean, and South Asian populations [3, 6].

G6PD deficiency is inherited in an X-linked fashion, being fully expressed in hemizygous males and homozygous females. In most cases, G6PD deficiency is asymptomatic. However, in some cases, acute hemolysis could be induced by oxidative stress, such as in hypoxia, bacterial or viral infection, or exposure to certain foods (e.g., Fava beans), chemicals, or drugs (e.g., quinolones/antimalarials) [7]. G6PD deficiency can also cause life-threatening hemolytic anemia during childhood with severe neonatal hyperbilirubinemia (NH) as the most fatal consequences of G6PD deficiency that can lead to chronic bilirubin encephalopathy (kernicterus) and spastic cerebral palsy [7].

G6PD deficiency and genetic variations (polymorphism and mutations) have been reported to be associated with hemoglobinuria or NH in several populations [6, 8]. Neonatal hyperbilirubinemia is quite common in Indonesia; however, it is not yet known whether *G6PD* genetic variants (polymorphisms and mutations) and deficiency are the risk factors for NH in the Indonesian population. Here we present a complete mutational and polymorphism analysis of *G6PD* and its activity in newborns with and without hyperbilirubinemia in Indonesian Deutromalay population.

## Methods

### Subjects

The subjects were healthy term neonates with hyperbilirubinemia (NH), defined as total serum bilirubin (TSB) above the 95th percentile for age in hours based on Bhutani’s nomogram. Neonates with TSB below the 40th percentile for age in hours based on Bhutani’s nomogram were included in this study as the control group. All neonates in both groups were single births. Neonates born to mothers with diabetes and those with neonatal sepsis, cephalohematoma, ABO or Rhesus blood group incompatibility with their mothers, or other congenital diseases which would affect the level of bilirubin in the serum were excluded. Two hundreds Seventy Six healthy term neonates from the Indonesian Deutromalay population were recruited consecutively from 5 hospitals in Sumatra and western part of Java islands. In total, only 116 and 115 neonates met the inclusion criteria for case and control groups, respectively, were included in this study. Written informed consent was obtained from parents, and the study was approved by the Ethics Committee of the Faculty of Medicine, Universitas Padjadjaran, Bandung, Indonesia. The characteristics of neonates and their mothers are presented in Table 1.

**Table 1:** Characteristic of Neonates and Mothers in Case and Control Groups

Characteristic	Cases		Controls		*p*-value*
	(*N* = 116)	%	(*N* = 115)	%	
Neonates					
Birth Weight (gram)					0.772
Average (SD)	3125 (345.6)	–	3138 (371.2)	–	
Range	2500–4300	–	2500–4250	–	
Feeding					0.483
Breast milk	94	81.0	98	85.3	
Formula	0	0	0	0	
Mix of both	22	19.0	17	14.7	
Sibling with Jaundice					0.319
Yes	26	22.4	19	16.4	
No	90	77.6	96	80.6	
ABO Blood Group	18	15.5	9	7.75	0.065
Coombs Test Results					
Negative	116		115		–
Positive	0		0		
Mothers					
Age (Years)					
Average (SD)	30 (6.1)	–	30.9 (5.8)	–	0.208
Parity					
1	54	46.6	41	35.7	0.063
Delivery method					
Cesarean delivery	57	49.1	87	75.7	< 0.001
Normal	53	45.7	28	24.3	
Vacuum	3	2.6	0	0	
Forceps	3	2.6	0	0	
Consanguinity					–
No	116	100	115	100	

*) Chi–square. except Birth weight

### Measurement of G6PD activity

Red blood cell (RBC) G6PD activity assays was performed in 231 neonates using a G6PD assay kit from Randox Laboratory LTD (PD410, United Kingdom) in triplicate for each sample. All G6PD activity assays were performed within 24 h of sample collection. Results of G6PD measurement were calculated in mUnits (U)/10^9^ erythrocytes. The G6PD enzyme activity was then converted into Units per g Hemoglobin (U/g Hb). The median value of G6PD activity of male in control group (13.10 U/g Hb) was used as the standard for 100% enzyme activity in this study. The results of G6PD activities were classified as deficiency when the enzyme activity was <30% and was considered as intermediate and normal when the activity was 30–80% and > 80%, respectively.

### DNA isolation

Genomic DNA was isolated from 300 μl of peripheral blood leukocytes using a DNA isolation kit (Roche Life Sciences) according to the manufacturer’s protocol. The DNA concentration was measured using a NanoDrop™2000 (Thermo Fisher Scientific).

### Deep-targeted next-generation sequencing (NGS) and data analysis

The *G6PD* gene was enriched with the *TruSeq Custom Amplicon* assay (Illumina, San Diego, USA) and oligos were designed using *Design Studio* (Illumina, San Diego, USA). Amplicons covered all exons and exon-intron boundaries with 10 bp on each end. The amplicons were sequenced using paired-end sequencing of 2 × 250 bps on a MiSeq (Illumina, San Diego, CA, USA). Data processing was performed as described in the previous study [9]. Variants that were not present in the database of Single Nucleotide Polymorphism 138 (dbSNP138) or had minor allele frequencies (MAFs) < 0.01 in the 1000 Genomes Project database were categorized as mutations (rare variants) and variants that were present in the dbSNP138 database with MAFs ≥0.01 in the 1000 Genomes Project database were categorized as polymorphisms (common variants).

### Association analysis of identified polymorphisms

Association analysis of polymorphisms with NH was performed using PLINK, an open-source whole-genome-association analysis tool set which can be used to perform a range of basic to large-scale association analyses of genotype/phenotype data [10]. Polymorphisms that were monomorphic had MAFs less than 0.05 or were missing in 95% all subjects were excluded from the association test. The false discovery rate was used for multiple testing to correct *P* values [11].

### Validation of *G6PD* mutations

Mutations identified by targeted deep sequencing using Next-Generation Sequencing (NGS) method on MiSeq (Illumina, San Diego, CA, USA) were validated using polymerase chain reaction (PCR) and Sanger sequencing. Validation was only performed when DNA was still available. The primers used to amplify target exons of *G6PD* were designed using Primer3 V.0.4.0 software (http://bioinfo.ut.ee/primer3-0.4.0/) and the primers sequences were presented in Additional file 1: Table S1. Polymerase Chain Reaction (PCR) and Sanger sequencing to validate the mutations identified by MiSeq were performed as described in our previous study [9].

### In silico analysis

The pathogenicity of identified *G6PD* mutations was predicted using Polymorphism Phenotyping V2 (PolyPhen-2) (http://genetics.bwh.harvard.edu/pph2/) and Mutation Taster® (http://www.mutationtaster.org/) program.

## Results

### Subjects characteristics

**Fig. 1:**
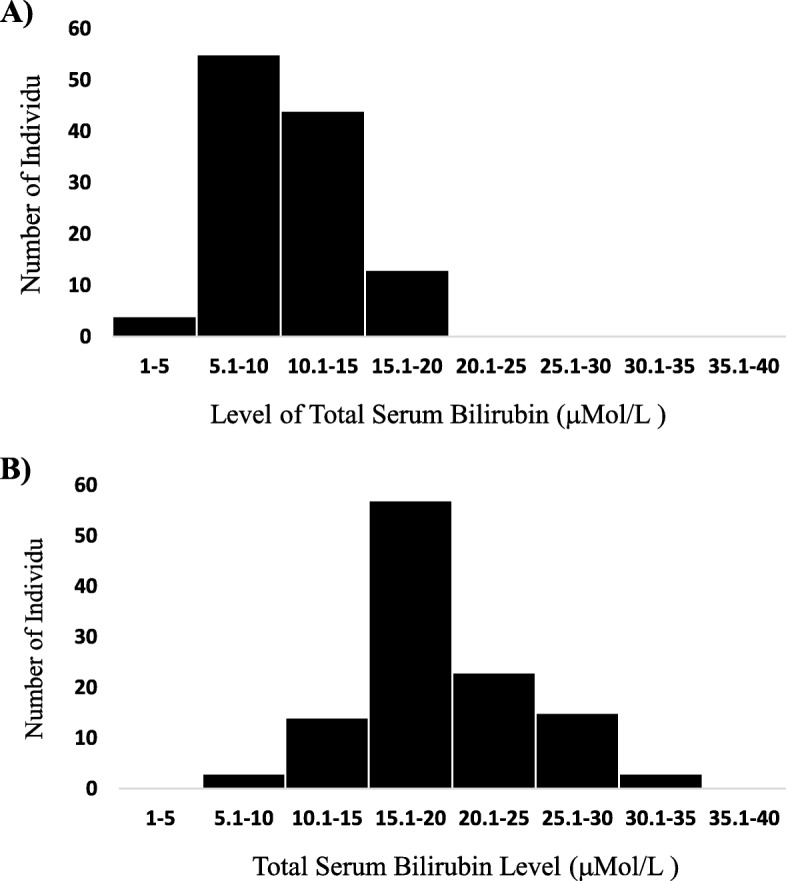
Histogram of Total Serum Bilirubin (TSB) level (μMol/L) distribution in: **a**) Neonates without hyperbilirubinemia (control group) and **b**) Neonates with hyperbilirubinemia (Cases group)

Although the difference was not significant, there were more boys (52.2–52.6%) than girls (47.4–47.8%) in both case and control groups. The mean birth weight was 3125 ± 345 g in the case group and 3138 ± 371 g in the control group. The results of ABO and Rhesus blood grouping incompatibility between mothers and neonates were negative in all samples (Table 1). There was no significant difference in feeding methods, maternal age, or parity between case and control groups. The majority of mothers in both groups had Cesarean delivery (57–87%) (Table 1). The histogram of total serum bilirubin (TSB) level distribution in case and control groups is presented in Fig. 1.

### The prevalence of G6PD deficiency

The prevalence of G6PD deficiency in case (2/116) and control (2/115) groups were 1.72% (95% CI: 0.6–4.1%) and 1.74% (95% CI: 0.7–4.1%), respectively.

### *G6PD* polymorphisms and association study

Nine *G6PD* polymorphisms were identified in cases and controls but only three of them (rs1050757/c.* + 357A > G, rs2230037/c.1311C > T, and rs2071429/c.1365-13 T/IVS11) had MAFs >0.05 in this population. Therefore, only those three polymorphisms and combinations of these variants (haplotypes) can be analyzed using PLINK. None of those polymorphisms and haplotypes were associated with NH in this population, as the *p*-value was >0.05 and the OR was ~1.00 (Table 2). In total, there were 4 G6PD-deficient subjects in case and control groups, but only 2 (50%) were identified with a combination of these three polymorphisms (Table 3).

**Table 2:** Association of G6PD Polymorphisms with NH

No.	SNP	Location	AA Change	Coordinate (Hg19)	Ref	F_A	F_U	Alt	P	OR	95% CI	q-value
1	rs1050757	Intronic	–	153,759,858	C	0.3000	0.2692	T	0.4626	1.163	0.66–2.06	0.6168
2	rs2230037	3UTR	–	153,760,654	A	0.2913	0.2735	G	0.6702	1.092	0.62–1.94	0.7468
3	rs2071429	Exon 11	p.Y437=	153,760,508	G	0.2913	0.2778	A	0.7468	1.069	0.60–1.89	0.7468

*AA* Amino Acid, *Alt* Alternative, *CI* Confidence of Interval, *F_A* Frequency Alt in Cases, *F_U* Frequency Alt in Controls, *Hg19* Human genome version 19, *OR* Odds Ratio, *Ref* Reference

**Table 3:** G6PD Activity, Mutations and Polymorphism Identified in Case and Control Groups

No.	Case (*N* = 6)							No.	Control (*N* = 9)						
	ID	F/M	*G6PD* Mutation	Genotype	G6PD Polymor-phism	G6PD Activity (U/g Hb)	G6PD Activity (%)		ID	F/M	*G6PD* Mutation	Genotype	G6PD Polymor-phism	G6PD Activity (U/g Hb)	G6PD Activity (%)
1.	21	F	p.V291 M	G/A	+	7.88	60.16	1.	202	M	–	–	–	0.24	1.84
2.	55	M	p.R459L	T/− (hem)	–	0.45	3.43	2.	175	M	p.V291 M	A/− (hem)	+	0.97	7.43
3.	80	F	p.V291 M	T/C	+	0.34	2.63	3.	19	F	p.V291 M	G/A	+	5.21	39.77
			p.L128P	G/A				4.	230	F	p.L128P	T/C	–	7.56	57.71
4.	229	M	p.R463H	A/− (hem)	–	10.65	81.29	5.	88	M	p.R459L	T/− (hem)	+	15.74	120.15
5.	101	M	p.V291 M	A/− (hem)	+	12.92	98.59	6.	181	M	p.L474 = #	A/− (hem)	+	13.71	104.66
6.	162	M	p.V369A# p.I167F#	C/− (hem) T/− (hem)	–	32.51	248.16	7.	184	M	p.I36T#	C/− (hem)	–	15.47	118.06
								8.	203	M	p.A335T	A/− (hem)	–	15.35	117.18
								9.	209	M	p.V291 M	A/− (hem)	+	12.91	98.54

#: novel mutation, F/M: Female/Male, hem:hemizygous, STB: Serum total bilirubin, G6PD polymorphism: rs1050757, rs2071429, rs2230037, G6PD deficient: G6PD activity <30%

### *G6PD* mutations and in silico analysis

The frequencies of mutations in the case and control groups were not significantly different. Eight *G6PD* mutations were identified in each group, hence the *G6PD* mutation frequencies in cases (8/116) and controls (8/115) were 6.8% (95% CI:2.3–11.5%) and 6.9% (95% CI:2.3–11.6%), respectively (Table 3). In total, from those neonates who carried *G6PD* mutations, nine different mutations identified in six and eight neonates from the case and control groups, respectively (Table 3). Among those mutations, two novel mutations were identified in both case and control groups. The two novel mutations identified in cases were all missense mutations (c.1106 T > C/p.V369A and c.499A > T/p.I167F), whereas in controls, one of them was a silent mutation (c.1422G > A/p.L474=) and the other was a missense mutation (c.107 T > C/p.I36T). The Viangchan variant (c.871G > A/ p.V291 M) was the most frequently identified mutation in both cases and controls (three neonates in each group), followed by the Canton variant (c.1376G > T/p.R459L) and Vanua Lava variant (c.383TA/p.L128P) which were identified in one neonate in each group. The Kaiping variant (c.1388G > A/p.R463H) was identified only in one case, and the Chatham variant (c.1003G > A/p.A335T) was identified only in one control (Table 3). The mutations identified in this population differ from those in previous studies in eastern Indonesian population, in which the predominant variant identified was the Vanua Lava variant [12, 13]. Among 4 G6PD-deficient neonates, two and one subjects in case and control groups, respectively, were identified as carriers of *G6PD* mutations. In the case group, one had the Canton Variant (p.R459L) and the other had a compound heterozygote with the Viangchan and Vanua Lava (p.L128P) variants (Table 3). In the control group, one subject was identified with the Viangchan variant, and the other did not have either *G6PD* mutation or polymorphism (Table 3). Hence, in this population, 75% (3/4) of G6PD-deficient neonates were carrying *G6PD* mutations. To predict the pathogenicity of identified mutations, in silico analysis using Polypen-2 and Mutation Taster® was performed. Most of the identified mutations, including the novel ones, were predicted to be disease-causing or possibly damage-causing variants (Additional file 1: Table S2). Functional studies on novel mutations are required to validate the results of in silico analysis.

### Validation of *G6PD* mutations

Among the nine different mutations identified in a total of 14 neonates (six in case group and eight control group), only five different mutations (56%) in seven neonates (50%) were validated by PCR and Sanger sequencing. Those were the Vanua Lava, Chatham, Canton, Viangchan, and Kaiping variants (Additional file 1: Figure S1-S2). Since the DNA samples of the other seven neonates were no longer available or the quality was significantly decreased, the mutations could not be validated by using Sanger sequencing.

## Discussion

The pathophysiology of NH is complex and multifactorial. Studies that have been conducted so far have focused on the prevalence of NH in G6PD-deficient neonates and shown that G6PD deficiency is one of the risk factors for NH in several populations or, at least, that G6PD-deficient neonates have significantly higher bilirubin levels than controls [8, 14]. To date, more than 150 *G6PD* variants have been identified as causal or risk factors in G6PD deficiency. Very limited studies have been performed to analyze *G6PD* variants (mutations and polymorphisms) in NH and the association of those variants with NH. One study has suggested that G6PD-deficient neonates carrying the c.563C > T *G6PD* variant developed jaundice earlier than infants without G6PD deficiency [15].

The results of this study show that the prevalence of G6PD deficiency in neonates with hyperbilirubinemia (NH) in this population (1.72%). This number is lower than the prevalence of G6PD deficiency in Malaysia (29.7%), Thailand (22.9%), Egypt (30.2–42%), Pakistan (17.3%) and India (2.5%) [1, 15–17]. The prevalence of G6PD deficiency in NH in this population was also lower than in the population infected by Malaria in eastern Indonesian population (Flores [4.4%] and Sumbawa [3.1–6.7%]) [12, 13]. Furthermore, the prevalence of G6PD deficiency in case group (1.72%) was similar to that in control group (1.74%) (Table 3). This shows that G6PD deficiency is not a major risk factor in the etiology of NH in this population.

Variant analysis using targeted deep sequencing allows us to accurately genotype all mutations and polymorphisms in the exonic regions, their flanking sites, and small parts of the upstream (3′-UTR) and downstream (5′-UTR) regions of the gene. Three polymorphisms (rs1050757, rs2230037, and rs2071429) with MAF >0.05% were identified in cases and controls. Among those polymorphisms, only one was located in the coding region: rs2071429 (p.Y437=). This variant is a silent polymorphism with frequencies of 29% (0.2913) in NH and 28% (0.2778) in controls (Table 2). Association analysis using PLINK showed that neither of these polymorphisms nor their combination (haplotype) were associated with NH. This study has a limitation in the form of significantly different cesarean-section rates in case and control groups (Table 1). This could introduce bias to the results of this study as the cesarean-section itself has been reported in the previous studies to be associated with hyperbilirubinemia [18, 19].

In total, there were 6 and 8 neonates from the case and control groups, respectively, were identified as carriers of *G6PD* mutations. However, despite of carrying *G6PD* mutations with or without polymorphism, only 2 neonates from each group suffer from G6PD deficiency. Three of those four G6PD-deficient neonates (75%) carry G6PD mutations with or without polymorphism and one of them does not have any *G6PD* coding mutation or polymorphism. This number is similar to those identified in Egyptian and Chinese population, where 62 and 78% of G6PD-deficient neonates, respectively, are identified as carriers of *G6PD* mutations, but lower than that in Singaporean (90%) and Pakistani population (94%) [14, 17, 20, 21]. Previous studies have shown that some variants or a haplotype in the non-coding region of *G6PD* (+357A > G/c.1365-13 T > C/c.1311C > T) were associated with the lower enzyme activity in individual without *G6PD* mutation in the coding region [21]. As in the deep-targeted sequencing method only small part of UTRs and intronic regions are included in the analysis, there is a possibility that we missed in identifying non-coding mutation in one of G6PD-deficient neonates.

G6PD deficiency is inherited in X-linked recessive pattern, hence this condition mostly affects male neonates. It is interesting to note that in this study, one of affected neonates is female. Her enzyme activity was only 2.63% of normal G6PD activity. This female neonate (ID-80) have compound heterozygous of Vianchan and Canton variants with polymorphism. This showing that gene dosage affects the severity of the enzyme deficiency. However, predicting the effect of a *G6PD* mutation in females is more complicated than in their male counterparts. Although the females have two X chromosomes, one X chromosome is randomly inactivated during embryogenesis. The total gene expression will depend on the ratio of the X-inactivated wild-type allele to the mutant allele. The remaining *G6PD* mutations were identified in male neonates, and most of them did not suffer from G6PD deficiency (Table 3).

Of all *G6PD* mutations identified in this study, the most common is the Viangchan variant (p.V291 M), followed by the Canton (p.R459L) and Vanua Lava (p.L128P) variants, which are located in exons 9, 12, and 5, respectively. The predominant mutational type identified in this study was different from those in other ethnic groups in Indonesia. A study in Sumba, eastern Indonesia, has shown that the predominant *G6PD* mutation identified in this ethnic group is Vanua Lava [12]. Indonesia is an archipelago country, with thousands of islands and more than 100 ethnic groups. It is not surprising that the predominant mutational type is different in each region in Indonesia. The ancestors of the Deutromalay, who inhabits the western part of Indonesia, are believed to have come from northern China who were migrated to Southeast Asia. Therefore, the variants identified in this ethnic group are similar to those in China, Thailand, and other Southeast Asian countries.

## Conclusion

The prevalence of G6PD deficiency is low in neonates with or without hyperbilirubinemia in Deutromalay Indonesian population. The *G6PD* mutations identified in this population are mostly similar to those identified in other South East Asian population.

## Supplementary information

**Additional file 1: Table S1**. Primers for Exons of *G6PD*, **Table S2**. in silico Analysis of *G6PD* Mutations Identified in Cases and Controls, **Figure S1.** A) Hemizygous Canton variant c.1376G > T/p.R459L was identified in one case (ID-55) and in one control (ID-88). B) Hemizygous Kaiping variant c.1388G > A/p.R463H was identified in one case (Male, ID-229). F: Female, M: Male, WT: Wild-type, **Figure S2.** A) Heterozygous Vanua Lava variant c.383 T > C/p.L128P were identified one case (Female, ID-80) and one control (Female, ID-230). B) Hemizygous Chatham variant c.1003GT > A/p.A335T was identified one case (male, ID- 203). C) Viangchan variant was identified in three cases and three controls. F: Female, M: Male, WT: Wild-type.

## Data Availability

Not applicable.

## Abbreviations

bp: base pairs
CI: Confidence interval
DNA: Deoxyribose nucleic acid
G6PD: *Glucose-6-Phosphage Dehydrogenase*
Hb: Hemogblobin
Kb: kilobase pairs
MAF: Minor allele frequency
NAD: Nicotinamide adenine dinucleotide
NADPH: Nicotinamide adenine dinucleotide phosphate hydrogen
NGS: Next-Generation Sequencing
OR: Odds ratio
PCR: Polymerase chain reaction
Polyphen-2: Polymorphism phenotyping v2
RBC: Red blood cells
SNP: Single nucleotide polymorphism
TSB: Total serum bilirubin
U: Unit
