# Supplementary material for: G6PD genetic variations in neonatal Hyperbilirubinemia in Indonesian Deutromalay population
Source: BMC Pediatr. 2019 Dec 20;19:506. doi: 10.1186/s12887-019-1882-z (PMC6923888; doi:10.1186/s12887-019-1882-z)
Supplement: Supplementary file 1 — Additional file 1: Table S1. Primers for Exons of G6PD, Table S2. in silico Analysis of G6PD Mutations Identified in Cases and Controls, Figure S1. A) Hemizygous Canton variant c.1376G > T/p.R459L was identified in one case (ID-55) and in one control (ID-88). B) Hemizygous Kaiping variant c.1388G > A/p.R463H was identified in one case (Male, ID-229). F: Female, M: Male, WT: Wild-type, Figure S2. A) Heterozygous Vanua Lava variant c.383 T > C/p.L128P were identified one case (Female, ID-80) and one control (Female, ID-230). B) Hemizygous Chatham variant c.1003GT > A/p.A335T was identified one case (male, ID- 203). C) Viangchan variant was identified in three cases and three controls. F: Female, M: Male, WT: Wild-type. [file 12887_2019_1882_MOESM1_ESM.docx]

**SUPPLEMENTARY**

| **Table S1** - Primers for Exons of *G6PD* | | |  |  |
| --- | --- | --- | --- | --- |
| **No** | **Primer Name** | **Sequence 5' --> 3'** | **Primer length (bp)** | **product size (bp)** |
| 1 | G6PD_Ex2_F | AGGGGCTAACTTCTCAATGC | 20 | 295 |
| 2 | G6PD_Ex2_R | CAACTTAGCAGAGCCTGTGG | 20 |  |
| 3 | G6PD_Ex5_F | GTCTGTCTGTCCGTGTCTCC | 20 | 404 |
| 4 | G6PD_Ex5_R | GGTGTTTCGTGGAGCAACG | 19 |  |
| 5 | G6PD_Ex6_F | GAGGTTCTGGCCTCTACTCC | 20 | 375 |
| 6 | G6PD_Ex6_R | CACCCTTGTCTGAGTTCTGG | 20 |  |
| 7 | G6PD_Ex9_F | AACTCAACACCCAAGGAGCC | 20 | 343 |
| 8 | G6PD_Ex9_R | CAGCTCTCTCAGGGTGTGG | 19 |  |
| 9 | G6PD_Ex10_F | GTCCACACCCTGAGAGAGC | 19 | 387 |
| 10 | G6PD_Ex10_R | TGAGGGAGAGAGTGTCTTGC | 20 |  |
| 11 | G6PD_Ex11_F | ATGATGACCAAGAAGCCGGG | 20 | 405 |
| 12 | G6PD_Ex11_R | GCAGTGGGGTGAAAATACGC | 20 |  |
| 13 | G6PD_Ex12_F | GCTATGGGGTGGCCTTTGC | 19 | 446 |
| 14 | G6PD_Ex12_R | CAGCTGAGGTCAATGGTCCC | 20 |  |

| **Table S2 –** *in silico* Analysis of *G6PD* Mutations Identified in Cases and Controls | | | | | | |
| --- | --- | --- | --- | --- | --- | --- |
| **No.** | **Nucleotide Change** | **AA Change** | **Exon** | **SNP** | ***In Silico* Analysis** | |
|  |  |  |  |  | **Mutation Taster** | **PolyPhen-2** |
| 1. | c.1376G>T | p.R459L (Canton) | 12 | rs72554665 | DC | SD |
| 2. | c.1388G>A | p.R463H (Kaiping) | 12 | rs72554664 | DC | BD |
| 3. | c.383T>C | p.L128P (Vanua Lava) | 5 | rs78365220 | DC | BD |
| 4. | c.1106T>C | p.V369A# | 10 | - | DC | SD |
| 5. | c.499A>T | p.I167F# | 6 | - | DC | SD |
| 6. | c.871G>A | p.V291M (Viangchan) | 9 | rs137852327 | DC | BD |
| 7. | c.1003G>A | p.A335T (Chantham) | 9 | rs5030869 | DC | BD |
| 8. | c.1422G>A | p.L474(=)# | 12 | **-** | DC | - |
| 9. | c.107T>C | p.I36T# | 2 | **-** | DC | BD |
| AA: Amino Acid. DC: Disease Causing. SD: Possibly Damaging. BD: Probably Damaging. B:Benign | | | | | | |

c.1376G>T/p.R459L


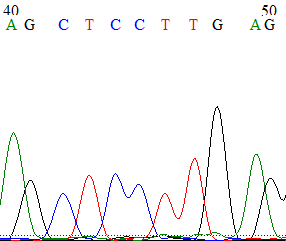

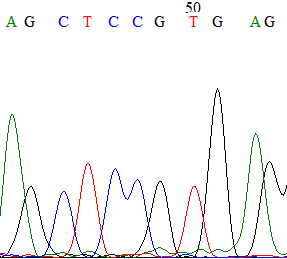


G/G

T/-


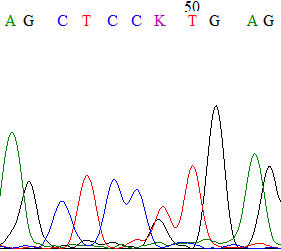


G/T

Control (F)

Homozygous WT

Control (F)

Heterozygous G/T

Patient (M)

Hemizygous

T/-

**A)**


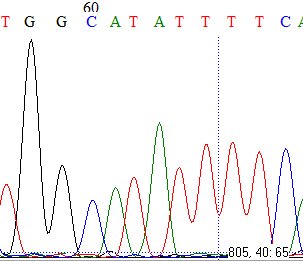


c.1388G>A/p.R463H


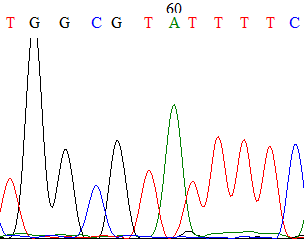


G/G

A/-

Control (F)

Homozygous WT

Patient (M)

Hemizygous A/-

**B)**

**Figure S1** – A) Hemizygous Canton variant c.1376G>T/p.R459L was identified in one case (ID-55) and in one control (ID-88). B) Hemizygous Kaiping variant c.1388G>A/p.R463H was identified in one case (Male, ID-229). F: Female, M: Male, WT: Wild-type.


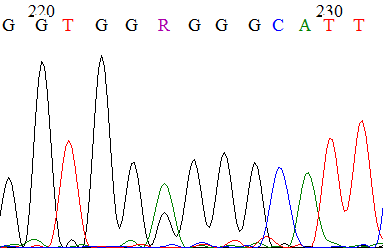


c.383T>C/p.L128P

A/G


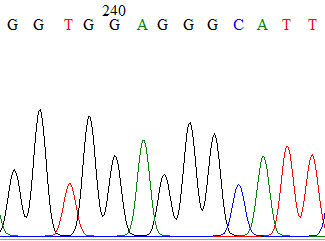


A/A

Control (F)

Homozygous

WT

Patient (F)

Heterozygous

A/G

**A)**

c.1003G>A /p.A335T

Control (M)

Hemizygous

WT


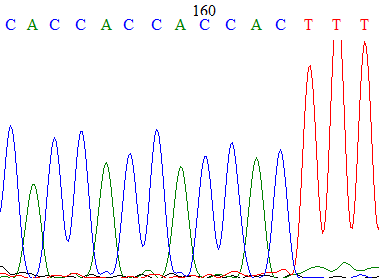

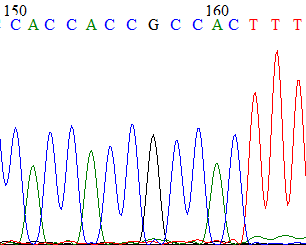


G/-

A/-

Patient (M)

Hemizygous

A/-

**B)**

**C)**


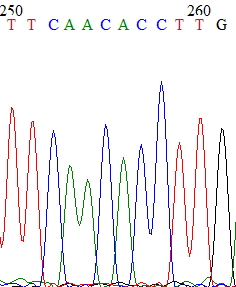


Patient (M)

Hemizygous

C/-

C/-

c.871G>A/ p.V291M (reverse seq)

**Figure S2** – A) Heterozygous Vanua Lava variant c.383T>C/p.L128P were identified one case (Female, ID-80) and one control (Female, ID-230). B) Hemizygous Chatham variant c.1003GT>A/p.A335T was identified one case (male, ID- 203). C) Viangchan variant was identified in three cases and three controls. F: Female, M: Male, WT: Wild-type
